# Fluorescence mechanism of poly(2-vinylnaphthalene) driven by local ordering and restricted motion

**DOI:** 10.1039/d6ra01104g

**Published:** 2026-05-01

**Authors:** Qiannan Zhang, Pingchuan Sun, Baohui Li

**Affiliations:** a School of Physics, Nankai University Tianjin 300071 P. R. China baohui@nankai.edu.cn; b Key Laboratory of Functional Polymer Materials of Ministry of Education and College of Chemistry, Nankai University Tianjin 300071 P. R. China spclbh@nankai.edu.cn

## Abstract

The fluorescence mechanism of poly(2-vinylnaphthalene) (P2VN), exhibiting aggregation-induced emission (AIE) characteristics, is systematically investigated through a combination of theoretical calculations and experimental characterizations. Density functional theory (DFT) calculations reveal that, as a π-electron-rich AIE-active polymer, the emission of P2VN originates primarily from the locally ordered stacking of its naphthalene side groups. This ordered arrangement enhances through-space interactions (TSI), which promotes exciton delocalization and significantly increases the fluorescence intensity. Solid-state NMR studies further demonstrate that the restricted molecular motion of naphthalene units is another key factor governing the AIE behavior as it effectively suppresses non-radiative decay and thus improves emission efficiency and intensity. This work elucidates the dual roles of through-space interactions and restricted intramolecular motion in achieving high-efficiency luminescence, providing deeper insights into the AIE mechanism of fluorescent polymers. The findings offer a theoretical foundation for the design of novel luminescent materials beyond traditional conjugated systems.

## Abstract 2

The fluorescence mechanism of poly(2-vinylnaphthalene) (P2VN), exhibiting aggregation-induced emission (AIE) characteristics, is systematically investigated through a combination of theoretical calculations and experimental characterizations.

## Graphical abstract

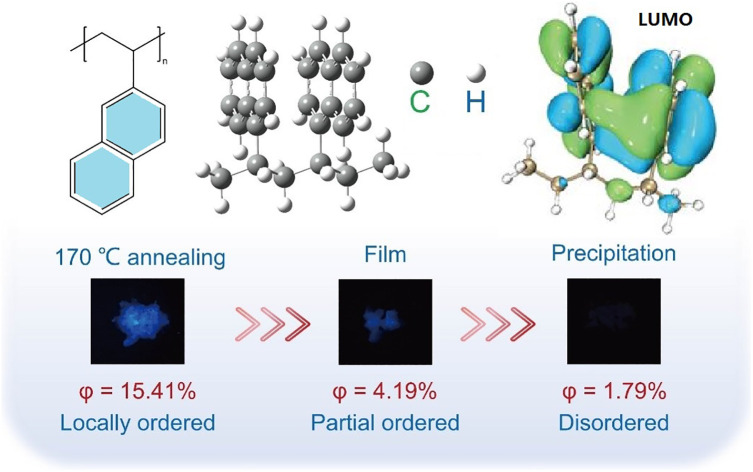

## 1 Introduction

In recent years, photoluminescent polymers with aggregation-induced emission (AIE) characteristics have attracted growing research attention due to their intense luminescence upon aggregate formation and potential applications in optoelectronics,^1–3^ sensing,^4–6^ encryption,^7–9^ chemical probes,^10–12^ and bioimaging.^13–15^ Most traditional organic luminescent polymers consist of conjugated systems in which their emissive units are interconnected *via* double bonds, triple bonds, heteroatoms, or aromatic rings.^16–19^ However, their inherent property limitations constrain their scope of application. Non-main-chain conjugated AIE polymers have become a research hotspot due to their high molecular weight, excellent processability, and film-forming properties. This category encompasses two representative systems: side-chain polymers with grafted aromatic rings and unconventional luminescent polymer systems exhibiting clusteroluminescence phenomena.^20–23^ Unlike conventional conjugated luminescence, the defining characteristic of non-traditional luminescent polymers is that their emission does not rely on a continuous π-conjugated system linked by covalent bonds. Instead, emission is dominated by aggregates formed *via* noncovalent interactions among spatially adjacent chromophores, making them an important research direction in the field of organic luminescence. Notable examples of unconventional luminescent polymers include natural products^24–28^ and synthetic polymers, such as polystyrene,^29–31^ polyurethane,^32–35^ and polypeptides.^36–38^ The emission of AIE polymers is activated upon aggregation, where noncovalent interactions among spatially adjacent chromophores restrict molecular motion and enhance radiative decay, positioning AIE polymers as a key focus in the field of organic luminescence.

Although these luminescent phenomena have been documented for decades, their underlying mechanisms remain poorly understood and cannot be adequately explained by classical photophysical theories. Recent studies have revealed that through-space interactions (TSI) are a common feature in AIE-active materials, including clusteroluminescent polymers, where spatially proximate aromatic groups enable electronic coupling and exciton delocalization without covalent linkages.^39–41^ Further investigations have demonstrated that electron coupling and delocalization play a pivotal role in the photoluminescence of such systems.^42–46^ For instance, 1,1,2,2-tetraphenylethane, in which phenyl rings are spatially separated by saturated carbon bridges, exhibits sky-blue emission in the solid state due to the excited-state electronic overlap between adjacent phenyl rings.^47^ Despite these advances, the current research on TSI remains largely qualitative, and its quantum mechanical underpinnings remain unresolved. Critical questions persist regarding the photophysical nature of TSI, its spatial manifestation, and its precise role in photon absorption and emission processes.

Herein, we investigate poly(2-vinylnaphthalene) (P2VN), a representative AIE-active polymer with abundant π-electrons, which exhibits unusual luminescent behaviour despite its simple molecular architecture of covalently linked naphthalene units. To date, no in-depth, comprehensive investigation has been conducted to elucidate the origin of its photophysical properties. Using P2VN as a model system, this work systematically unveils the microscopic mechanism of its photoluminescence by integrating spectroscopic analysis, density functional theory (DFT) simulations, and solid-state nuclear magnetic resonance (NMR) techniques. Specifically, DFT calculations focus on spatial interactions, electronic coupling, and exciton delocalization between naphthalene rings, particularly the differences in fluorescence emission mechanisms among various locally ordered regions. Solid-state NMR is employed to investigate the influence of the molecular motion of naphthalene rings on luminescence behavior. This study aims to deepen the understanding of fundamental AIE mechanisms in non-conjugated backbone polymer systems and to provide a theoretical basis for the rational design of novel AIE-active polymers with tailored photophysical properties.

## 2 Experiment and methods

### 2.1 Materials

All chemicals and reagents were obtained from commercial sources. Poly(2-vinylnaphthalene) (P2VN) was purchased from J&K Scientific Ltd (China). All the solvents and chemicals were used without any further purification or treatment. Nuclear magnetic resonance (NMR) spectra and molecular weight distribution of the polymer are presented in the SI (Fig. S2).

### 2.2 Preparation of random, Loosely Ordered and partially ordered P2VN

Three P2VN samples with distinct naphthalene-ring stacking structures, designated as PV1, PV2, and PV3, were prepared *via* three different processing methods: annealing at 170 °C, film formation from tetrahydrofuran (THF), and methanol precipitation, respectively.

Annealing treatment (PV1-partially ordered P2VN samples):

To induce structural ordering, the P2VN sample was annealed at 170 °C for 15 minutes, followed by controlled cooling to room temperature. During the annealing treatment, the heating rate was 15 °C min^−1^, and annealing was performed under a nitrogen atmosphere with a flow rate of 10 mL min^−1^. This process promoted tighter molecular packing, resulting in a partially ordered spatial arrangement.

Solvent evaporation method (PV2-loosely ordered P2VN thin film):

P2VN was dissolved in THF, and the resulting solution was cast onto a substrate. Slow solvent evaporation at ambient conditions, followed by thorough drying, produced a loosely packed thin film with partial spatial ordering.

Precipitation method (PV3-random P2VN samples):

A homogeneous solution of P2VN was prepared by dissolving the polymer in tetrahydrofuran (THF), a good solvent. The concentration of P2VN in tetrahydrofuran (THF) was 10 mg mL^−1^. The addition rate into the poor solvent was 3.0 mL min^−1^. This solution was then slowly introduced *via* a pipette into an excess of poor solvent under gentle stirring. After complete precipitation, the poor solvent was removed, yielding a randomly structured P2VN sample.

### 2.3 Photoluminescence measurements

Steady-state photoluminescence spectra and quantum yields (solid state) were acquired using an Edinburgh Instruments FS5 spectrofluorometer equipped with a 450 W xenon arc lamp and µs flashlamp. Excitation wavelengths were set at 280, 300, 320, 340, and 360 nm.

### 2.4 Solid-state UV-vis spectroscopy measurements

UV-vis absorption spectra (200–500 nm) were recorded on a Shimadzu UV-2600 spectrophotometer in absorbance mode using solid sample holders.

### 2.5 X-ray diffraction analysis

Wide-angle X-ray diffraction (XRD) patterns (2*θ* = 0°–80°) were obtained using a Rigaku SmartLab diffractometer (Japan) with Cu Kα radiation (*λ* = 1.5406 Å). Samples were mounted on a zero-background silicon holder and scanned over a 2*θ* range of 0°–80° with a step size of 0.02° and a counting time of 1 s per step.

Small-angle X-ray scattering (SAXS) measurements (0–4 Å^−1^*q*-range) were performed on a Xenocs Xeuss 3.0 system (France) equipped with a GeniX 3D microfocus source (Cu Kα, *λ* = 1.5406 Å) and a Pilatus 1 M detector. Samples were loaded in a dedicated sample holder and measured in transmission mode, covering a *q*-range of 0–4 Å^−1^ (corresponding to *d*-spacings from ∞ to ∼1.5 Å), where *q* = (4π sin *θ*)/*λ*.

### 2.6 Solid-state nuclear magnetic resonance (NMR) measurements

All solid-state NMR experiments were performed on a Varian InfinityPlus-400 spectrometer at a proton resonance frequency of 400 MHz. The sample was packed into a 52 µL zirconia pencil-type rotor, and magic-angle spinning (MAS) was applied for all one- and two-dimensional experiments. The internal controller of the instrument automatically regulated the spinning speed to 9.8 kHz ± 2 Hz.

### 2.7 Theoretical calculations

All calculations were performed using the Gaussian 16 and Multiwfn software packages.^48,49^ Ground-state geometries were optimized at the B3LYP/6-31G(d) level with Grimme's DFT-D3 dispersion correction. Frequency analyses confirmed the presence of a local minimum on the potential energy surfaces. Excited states were calculated using time-dependent DFT (TD- DFT) at the same level of theory. The polymer system was modelled using an oligomer approximation method with benzene repeat units ranging from monomeric (single ring) to pentameric (five rings).

## 3 Results and discussion

### 3.1 Microstructure characterization of P2VN

To elucidate the microscopic mechanism of fluorescence emission in P2VN, X-ray diffraction (XRD) and small-angle X-ray scattering (SAXS) measurements were performed on samples obtained under different processing conditions. As illustrated in Fig. 2A, all three samples exhibited broad diffraction peaks in the 2*θ* range of 10°–40°, indicating an overall amorphous nature of the P2VN materials. The peak center at approximately 20° corresponds to a naphthalene ring stacking distance of about 4.7 Å. Notably, the diffraction peak of the PV1 sample annealed at 170 °C is relatively sharper and more intense, suggesting enhanced local ordering. This thermal annealing treatment improves photoluminescence performance by increasing intermolecular order.

SAXS results (Fig. 2B) show that the relative intensity ratio of the two scattering peaks at 1.26 nm^−1^ and 0.4 nm^−1^ increases sequentially for PV1, PV2, and PV3. This indicates that thermal annealing enhances the long-range order of the naphthalene stacking structure. The improved ordering strengthens intermolecular interactions between naphthalene rings, thereby promoting higher luminescence intensity. Additionally, P2VN exhibits strong ultraviolet absorption around 310 nm, which originates from the π–π* electronic transition of its π-electron-rich structure.

### 3.2 Photoluminescence (PL) properties of P2VN

**Fig. 1:**
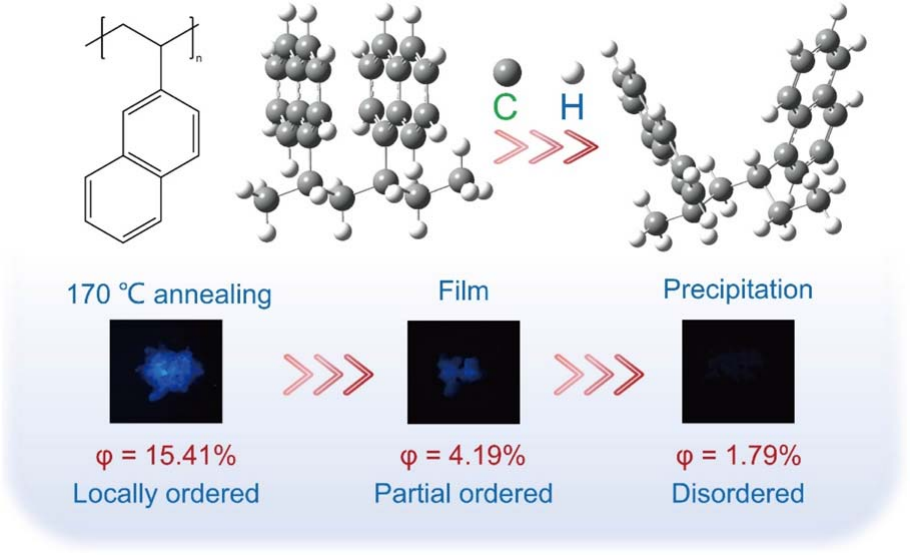
Characterization of P2VN exhibiting local order-disorder structures *via* annealing, film formation, and precipitation methods under 365 nm UV irradiation.

P2VN samples, designated as PV1, PV2, and PV3, were systematically prepared *via* three distinct processing routes: annealing, film formation, and precipitation. Under the same excitation wavelength, the semi-crystalline sample PV1, which forms a locally ordered structure induced by thermal annealing at 170 °C, exhibits a photoluminescence quantum yield (PLQY) of up to 15.41%. In contrast, the fluorescence of sample PV2 is weaker, while PV3 shows almost no fluorescence (Fig. 1). As illustrated in Fig. 3A–C, the UV/vis absorption spectra and excitation spectra of these samples demonstrate a high degree of consistency. PV1 displays a distinct absorption peak near 290 nm, attributed to the π–π* electronic transition of naphthalene rings, indicating that the enhancement of π–π* transitions is closely related to the specific aggregation structure of naphthalene rings.^50^ By comparison, the film-state sample PV2 shows a significant absorption peak near 315 nm, suggesting a red shift in absorption and a change in the stacking structure of naphthalene rings. The experimentally measured solid-state UV-vis spectra are in good agreement with the theoretically simulated spectra (SI Fig. S4B) in terms of major spectral features, strongly confirming the reliability of experimental and computational methods, with only minor deviations in peak intensity or absorption edge position. The absorption peak of the precipitated P2VN is less pronounced and shifted to the right, signifying a decrease in the degree of aggregation of the naphthalene ring. We performed theoretical calculations by constructing P2VN models with ordered and random stacking configurations and simulating their absorption spectra (SI Fig. S5A). The ordered stacking model exhibits a characteristic narrow peak, while the random stacking model yields a broadened peak, consistent with our experimental solid-state absorption spectra (Fig. 3A–C). The calculated HOMO–LUMO gap decreases from 4.50 eV (ordered stacking) to 4.26 eV (random stacking, 4.26 < 4.50), aligning with the observed red-shift trend. These results confirm that the wavelength shift and peak broadening of P2VN arise from stacking structure differences induced by varied preparation processes.

**Fig. 2:**
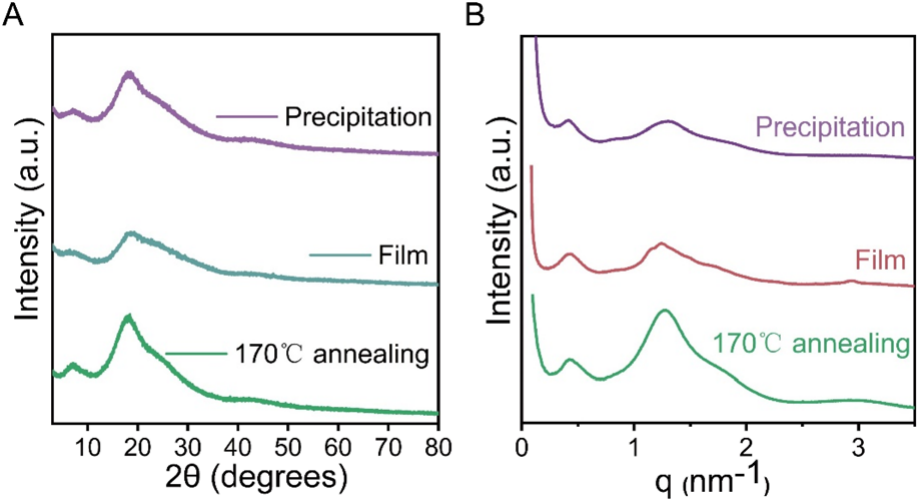
(A) XRD spectra of the annealed, film-layered, and precipitated samples at 170 °C. (B) SAXS spectra of the annealed, film-layered and precipitated samples at 170 °C.

**Fig. 3:**
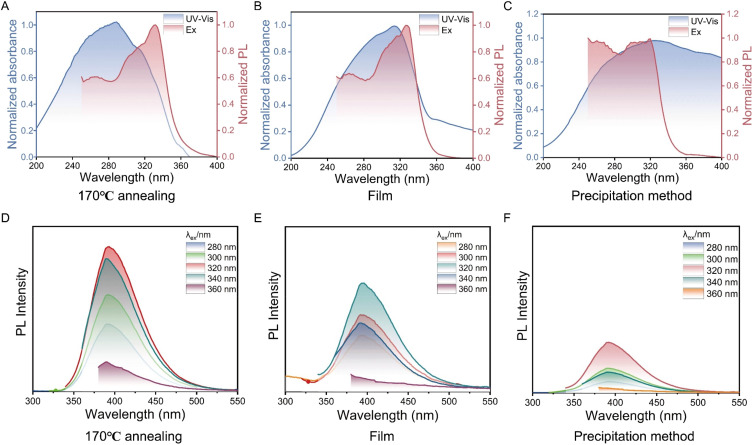
Normalized UV/vis absorption and excitation spectra of the samples prepared under different conditions: (A) annealing at 170 °C, (B) spin-coated film, and (C) precipitated solid. Photoluminescence (PL) spectra of the samples subjected to three different processing methods: (D) 170 °C annealing, € film casting, and (F) precipitation.

To elucidate the mechanistic origin of this enhanced emission, we conducted photoluminescence spectroscopy on three types of samples. By testing with excitation light of varying wavelengths (280 nm, 300 nm, 320 nm, 340 nm, and 360 nm), the emission wavelengths of all samples, which were consistently observed at 392 nm, are shown in Fig. 3D–F. The optimal excitation wavelength was found to be 320 nm, consistent with the results from the UV-vis absorption spectra. The experimental observation that the emission wavelength of P2VN is independent of the excitation wavelength suggests that the naphthalene rings in P2VN serve as fixed intrinsic luminescent centers.

### 3.3 Through-space interactions (TSI) of the naphthalene rings in poly(2-vinylnaphthalene) luminescence

**Fig. 4:**
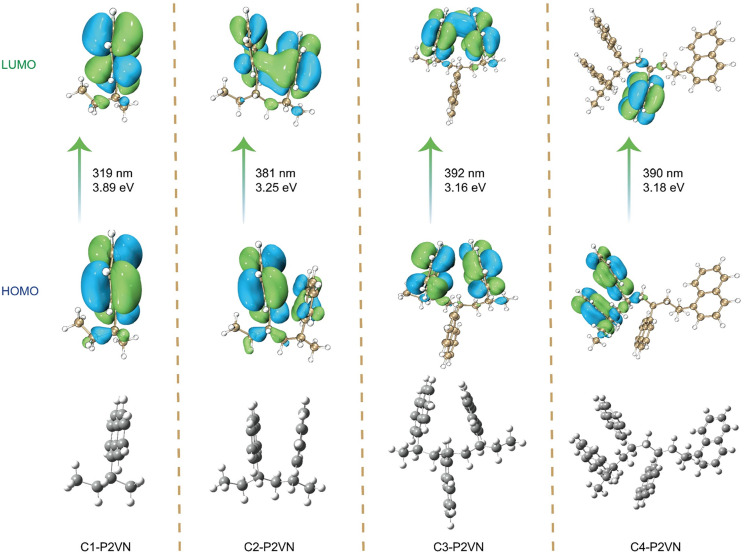
Natural transition orbitals (NTOs) for the gas-phase optimized excited state.

Building upon these foundational studies, we systematically investigated the TSI between adjacent naphthalene rings in the samples of P2VN and their influence on luminescent properties. The investigation focused on elucidating the origin of excited-state through-space interactions (ESTSI) that emerge upon modifying the number and spatial arrangement of naphthalene rings within the carbon backbone. To accurately simulate the through-space interactions (TSI) between the naphthyl side groups in P2VN, oligomer models with different number of naphthalene rings (C1-P2VN to C4-P2VN, Fig. 4) were constructed using the oligomer approximation method. This approach represents a classic and reliable strategy for simulating the photophysical properties of polymers. The model molecules were designed based on the repeating unit of P2VN (–CH_2_–CH (naphthalene)–). The backbones of oligomers with 1 to 4 repeating units were truncated, and hydrogen saturation was applied at the terminal positions to eliminate the end effects. The alkyl backbones of all model molecules were set in an all-trans conformation, and the dihedral angles between adjacent naphthalene rings were adjusted to a partially overlapped configuration. Through theoretical calculation of S_1_ excited states, we observed significant spatial overlap in the lowest unoccupied molecular orbitals (LUMOs). These ESTSI phenomena effectively stabilize the LUMOs and reduce the energy gap (Fig. 4). Calculations demonstrate that, under the condition of a fixed naphthalene ring spacing of 4.7 Å, the theoretical emission wavelengths of P2VN model compounds gradually increase from 381 nm to 392 nm and 390 nm as the number of adjacent naphthalene rings is raised from one to four. These results are in excellent agreement with the experimental fluorescence spectra (Fig. 2D–F). Furthermore, both the inter-naphthalene ring spacing and the dihedral angle between adjacent rings influence the luminescence efficiency. A significant enhancement in emission performance is observed only when the naphthalene rings are in a partially overlapping configuration, as this arrangement facilitates effective through-space interactions between the rings (Fig. S3 A and B).^51–53^

**Fig. 5:**
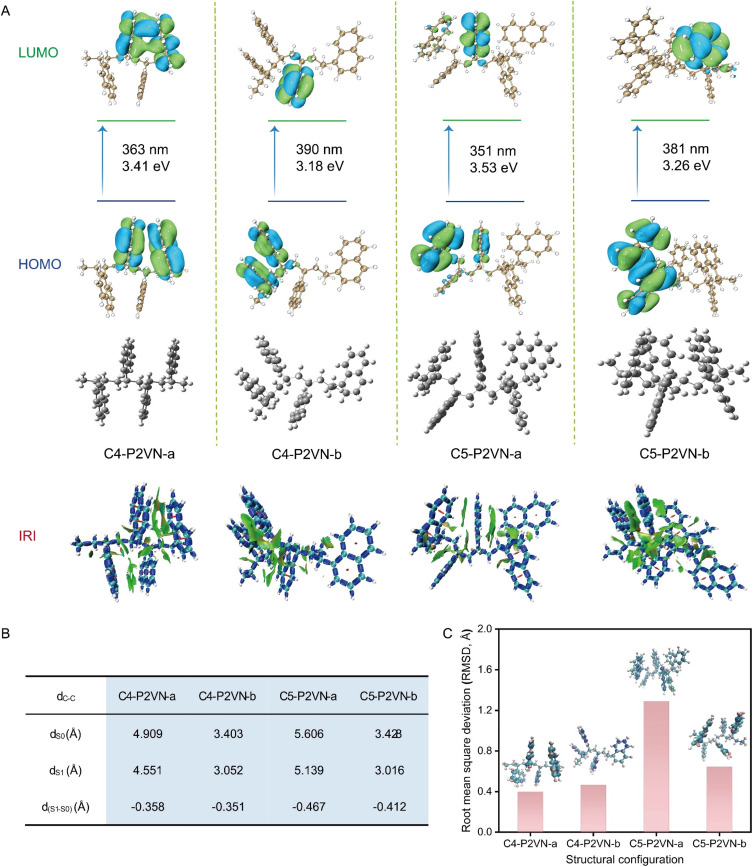
(A) NTO and interaction region indicator (IRI) isosurfaces for the gas-phase optimized excited states of four- and five-arm P2VN structures. (B) Changes in the carbon–carbon distances (*d*_C−C_) between side chains during geometric optimization from the ground state (dS_0_) to the excited state (dS_1_). (C) RMSD plots for P2VN with varying alkyl chain lengths.

The calculations further reveal that the fluorescence emission of P2VN exhibits a pronounced distance dependence. The model compounds are denoted as C*x*-P2VN-a, b, where *x* represents the number of naphthalene rings, and a and b indicate different conformations. When constructing C4-P2VN-a/b and C5-P2VN-a/b (Fig. 5A), the distances between all naphthalene rings were set to >4.7 Å (extended conformation, labeled a), while the distances between some naphthalene rings were set to <4.7 Å (compact conformation, labeled b) to investigate the distance dependence of TSI. The other structural parameters were consistent with the design rules described above. As shown in Fig. 5A, when the inter-ring spacing exceeds 4.7 Å (as in C4-P2VN-a and C5-P2VN-a), the calculated emission wavelengths are 363 nm and 351 nm, respectively, which deviate significantly from the experimental values. In contrast, the calculated emission wavelengths for the closely packed conformations with inter-ring spacings smaller than 4.7 Å (C4-P2VN-b and C5-P2VN-b) are 390 nm and 381 nm, respectively, showing excellent agreement with experimental data.

The inter-naphthalene-ring interactions were analyzed using the Interaction Region Indicator (IRI) isosurface. The isosurface appears predominantly green, which is a characteristic feature of van der Waals interactions (Fig. 5A, below). Notably, C4-P2VN-b and C5-P2VN-b display more extensive isosurface areas between adjacent naphthalene rings, indicating stronger intermolecular interactions. Fig. 5B demonstrates significant excited-state contraction of the closest inter-ring C–C distance (*d*(S_1_–S_0_)), while Fig. 5C presents the corresponding RMSD values. The RMSD reflects overall conformational changes. Notably, compared with the extended conformations (C4-P2VN-a and C5-P2VN-a), the compact conformations (C4-P2VN-b and C5-P2VN-b) exhibit stronger ESTSI and shorter spatial distances, thereby enhancing electron transition efficiency and leading to a red shift in the emission wavelength. These findings establish that ESTSI between proximal naphthalene units governs the bathochromic shift in the fluorescence emission of P2VN.

### 3.4 Analysis of orbital delocalization in P2VN molecules

**Fig. 6:**
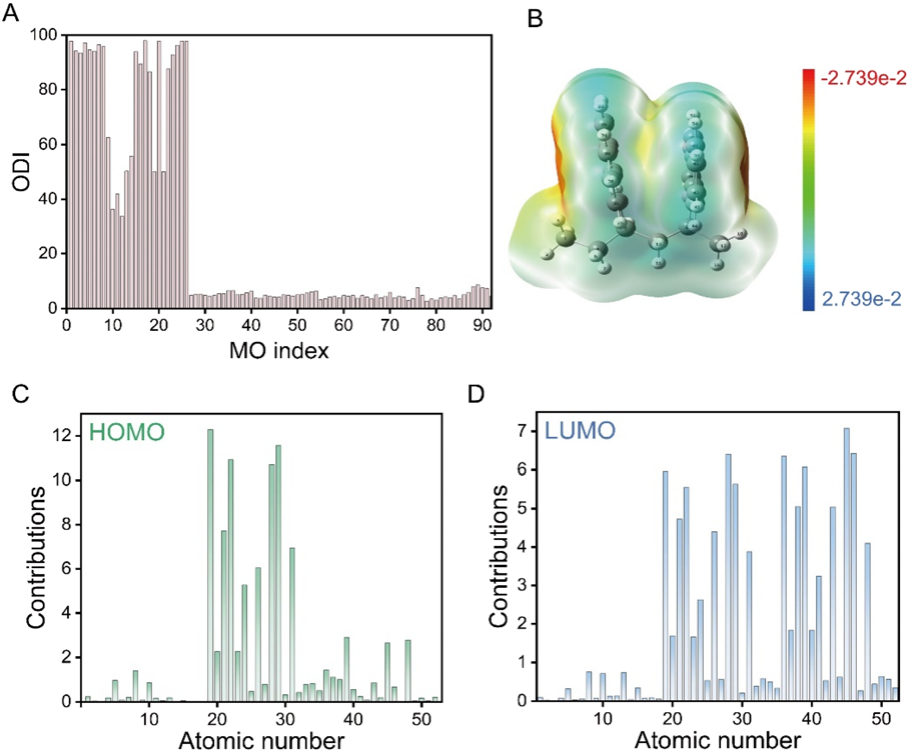
(A) Orbital delocalization indices (ODIs) with the corresponding molecular schematic of P2VN. (B) Electrostatic potential distribution on the molecular surface of P2VN. (C) Atomic contributions to the HOMO ODI in NTO analysis. (D) Atomic contributions to the LUMO ODI in NTO analysis.

Finally, using a dinaphthyl model system, we systematically analysed the orbital delocalization characteristics in P2VN. The extent of delocalization is quantitatively evaluated using the Orbital Delocalization Index (ODI), where lower ODI values indicate greater orbital delocalization. As shown in Fig. 6A, the result reveals that the first 26 orbitals primarily represent core electrons, consequently exhibiting relatively large ODI values (approximately 50% for certain orbitals, reflecting their localization within the core regions of two atomic centres). The theoretical calculation results demonstrate that both the highest occupied molecular orbital (HOMO) and the lowest unoccupied molecular orbital (LUMO) possess notably small ODI values, indicating strong delocalization. This characteristic facilitates efficient intermolecular electron transfer, thereby significantly enhancing molecular stability.

Detailed decomposition analysis shows that atoms 19, 21, 22, 24, 26, 28, 29, and 31 make predominant contributions to the ODI of the HOMO (Fig. 6C), while the ODI of the LUMO additionally incorporates significant contributions from atoms 36, 38, 39, 41, 43, 45, 46, and 48 (Fig. 6D). As illustrated in Fig. 6B, the molecular surface electrostatic potential analysis revealed that P2VN maintains an obvious electrostatic interaction. Notably, the outer non-interacting regions of the two naphthalene rings in P2VN display localized electronegativity, whereas the inner interacting region exhibits distinct electrical neutrality. This phenomenon indicates the presence of through-space interactions accompanied by charge transfer between the naphthalene rings, a finding consistent with the ODI analysis results. These findings provide clear evidence for through-space electron delocalization between naphthalene rings, offering fundamental theoretical support for enhancing luminescent properties *via* through-space interactions.

### 3.5 Effects of molecular motion on the luminescence properties of polymers

**Fig. 7:**
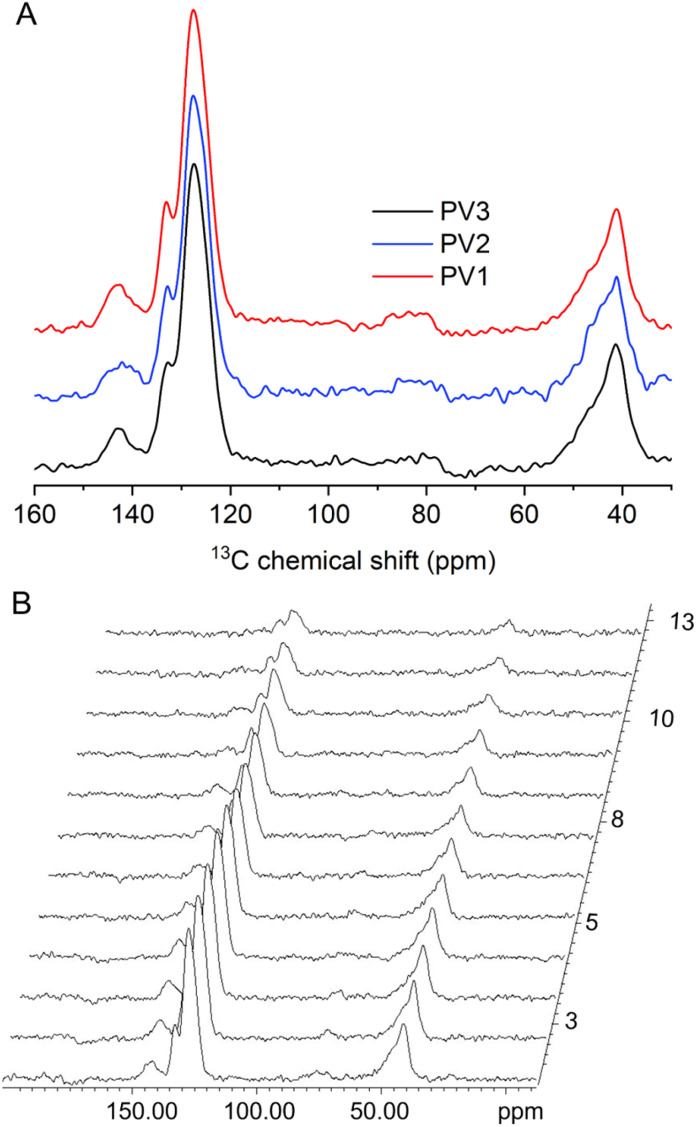
(A) ^13^C CPMAS spectra of PV1, PV2, and PV3. (B) Stacked spectra of the ^13^C spin-lattice relaxation time measurements with different delay times of PV1.

The luminescent properties of polymers are closely related to their molecular motion. Previous studies on AIE molecules have proposed microscopic mechanisms such as restriction of intramolecular rotation (RIR), restriction of intramolecular vibration (RIV), and restriction of intramolecular motion (RIM) to explain fluorescence emission: active molecular motion can dissipate exciton energy, while restricted molecular motion can activate radiative transitions. In this study, the spin–lattice relaxation times (*T*_1_^C^) of naphthalene ring luminescent centers in samples treated under different conditions were measured by solid-state NMR to investigate the relationship between the molecular motion of naphthalene rings and luminescent properties (Fig. 7). The measured *T*_1_^C^ relaxation time corresponds to aromatic carbon atoms of the naphthalene rings in the polymer.

Fig. 7A shows the ^13^C CPMAS spectra of the three samples, which exhibit no noticeable differences, indicating that the different treatment conditions did not alter the molecular structure of the polymer. The measured *T*_1_^C^ values for samples PV1, PV2, and PV3 are 47.6 s, 47.3 s, and 41.6 s, respectively. Fig. 7B displays the stacked spectra of PV1 obtained from spin–lattice relaxation experiments at different delay times. For solid polymer samples, a longer *T*_1_^C^ reflects slower molecular motion. Therefore, in the annealed sample PV1, the naphthalene rings are tightly packed, leading to restricted motion, which favors radiative transitions and results in the strongest luminescence. In contrast, the methanol-precipitated sample PV3 exhibits a less ordered packing structure of naphthalene rings, leading to faster molecular motion, enhanced non-radiative transitions, and dissipation of exciton energy, which explains its nearly negligible fluorescence. These results demonstrate that restricted molecular motion of luminescent centers is one of the key mechanisms for achieving high-efficiency luminescence in polymers.

### 3.6 Temperature-dependent photoluminescence analysis of annealed P2VN

**Fig. 8:**
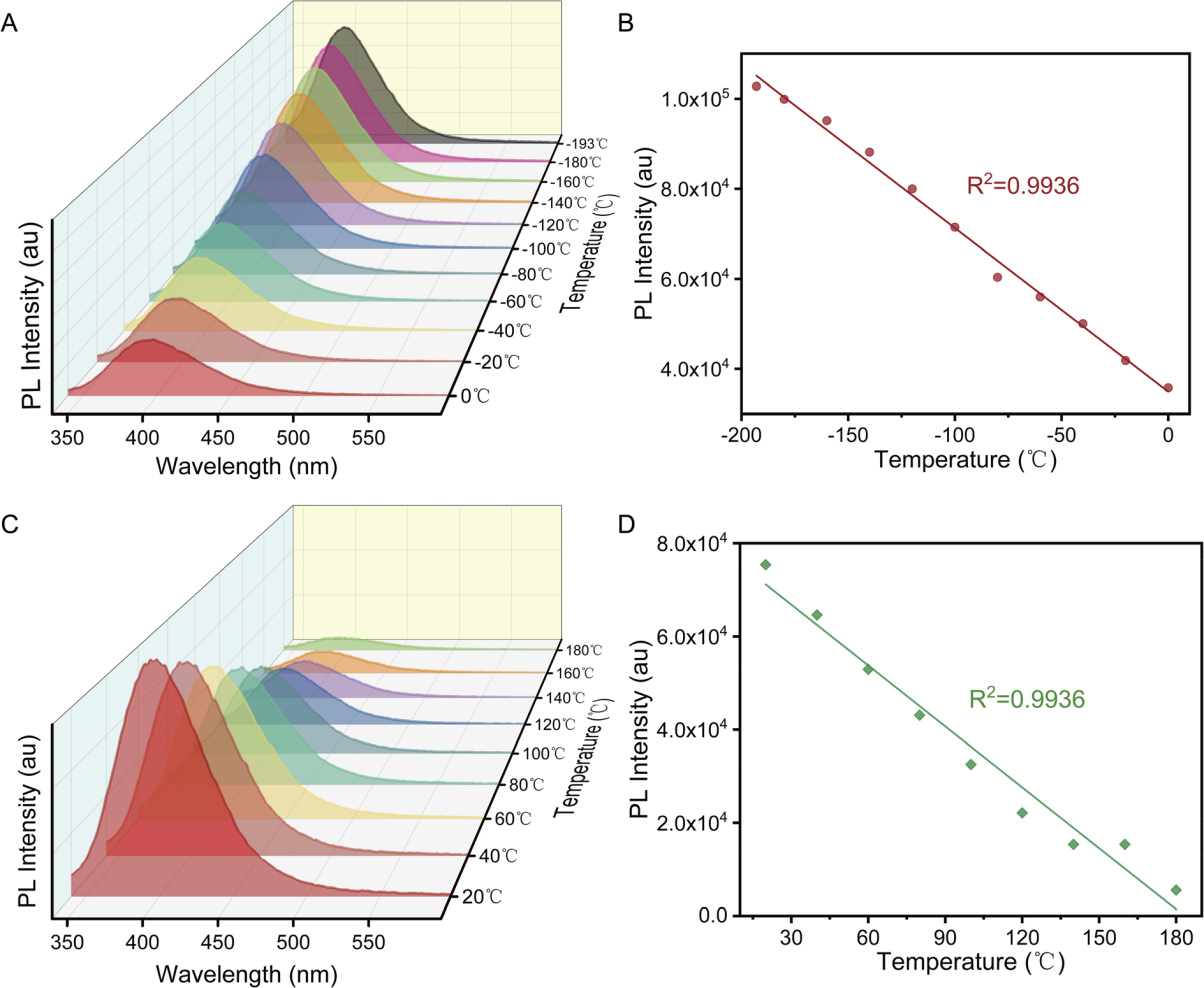
(A) Temperature-dependent photoluminescence (PL) intensity of P2VN in the low-temperature regime. (B) Linear fitting analysis of PL intensity as a function of low temperature. (C) Temperature-dependent PL intensity of P2VN in the high-temperature regime. (D) Linear fitting analysis of PL intensity as a function of high temperature.

The PL properties of the annealed sample PV1 were systematically investigated across different temperature regimes, with particular emphasis on the correlation between PL intensity and temperature (*T*) in both low-temperature (<273 K) and high-temperature (>273 K) ranges. Remarkably, the PL emission wavelength remained constant at approximately 398 nm under both high and low-temperature conditions, providing strong evidence for the exceptional structural stability of P2VN aggregates. A linear dependence of the PL intensity on temperature is observed in the low-temperature regime (Fig. 8A and B). The high-temperature-dependent PL measurements (Fig. 8C and D) also reveal a gradual reduction in PL intensity with increasing temperature, attributable to thermally activated intramolecular non-radiative processes. Similarly, the PL intensity exhibits a linear relationship with temperature in the high-temperature range. The observed high stability and linear temperature response of P2VN aggregates over a wide thermal range underscore their potential for applications in optoelectronic devices and temperature-sensing technologies.

## 4 Conclusions

In summary, this study comprehensively elucidates the influence of the aggregated-state structure and molecular motion on the luminescence properties of P2VN, as well as the microscopic mechanism underlying its AIE effect, through systematic quantum chemical theoretical calculations combined with solid-state nuclear magnetic resonance (NMR), spectroscopy, and other characterization techniques. Theoretical calculations confirm that the aggregation-induced emission behavior of P2VN is co-regulated by the spatially delocalized exciton coupling effect induced by the locally ordered stacking of naphthalene ring luminescent centers, through-space interactions (TSI) in the excited state, and restricted molecular motion. Experimental results demonstrate that adjusting material processing methods (such as thermal annealing) can effectively modulate the molecular packing order and motional freedom of the polymer, thereby significantly enhancing its luminescence performance. This work not only provides a novel perspective for understanding the microscopic origin of the AIE effect in luminescent polymers but also establishes a theoretical foundation for the rational design and development of advanced luminescent polymer materials.

## Author contributions

Qiannan Zhang: conceptualization, data curation, writing original draft, and visualization. Baohui Li: conceptualization, resources, and writing–review and editing. Pingchuan Sun: resources, writing–review and editing, supervision, project administration, and funding acquisition.

## Conflicts of interest

There are no conflicts to declare.

## Supplementary Material

RA-016-D6RA01104G-s001

## Data Availability

Data are available upon request. The data supporting this article have been included as part of the supplementary information (SI). Supplementary information: FT-IR spectra, theoretical calculation details.ion details, ^1^H-NMR spectra of P2VN in CDCl_3_ and GPC traces of P2VN. See DOI: https://doi.org/10.1039/d6ra01104g.
