# Supplementary material for: Fluorescence mechanism of poly(2-vinylnaphthalene) driven by local ordering and restricted motion
Source: RSC Adv. 2026 May 1;16(25):22775–83. doi: 10.1039/d6ra01104g (PMC13133857; doi:10.1039/d6ra01104g)
Supplement: RA-016-D6RA01104G-s001 [file RA-016-D6RA01104G-s001.pdf]

Supplementary Information (SI) for RSC Advances.
This journal is © The Royal Society of Chemistry 2026

Supporting Information

Fluorescence Mechanism in Poly(2-vinylnaphthalene) Driven by Local Ordering and Restricted Motion

Qiannan Zhang, [a] Pingchuan Sun*[b] and Baohui Li*[a].

1. *School of Physics, Nankai University, Tianjin 300071, P. R. China.*

2. *Key Laboratory of Functional Polymer Materials of Ministry of Education and College of Chemistry, Nankai University, Tianjin 300071, China.*

*To whom correspondence should be addressed: spclbh@nankai.edu.cn; baohui@nankai.edu.cn

Table of Contents

1 FT-IR Analysis of P2VN Samples 3

2 ^{1}H-NMR spectra of P2VN in $CDCl_3$ and GPC traces of P2VN 4

3 Theoretical Calculations 4

3.1 The scanning and characterization of the potential energy surface in the conformational space of P2VN 4

3.2 Theoretical analysis of P2VN molecular properties and the photoluminescence spectra of P2VN in THF solutions at different concentrations 6

1 FT-IR Analysis of P2VN Samples

Distinct C–H stretching vibrations were observed at 3046.4 cm^{-1} and 2917.5 cm^{-1}, consistent with the presence of vinyl groups in the polymer backbone. Additionally, peaks at 1268.1 cm^{-1} and 1120.6 cm^{-1} were assigned to C=C stretching modes within the naphthalene rings, while those at 1629.7 cm^{-1} and 1601.0 cm^{-1} arose from C-C stretching between adjacent naphthalene units. Comparative analysis revealed that the principal absorption features—particularly those associated with C-H and C-C bonding—remained consistent across all three processing methods. This spectral similarity indicates that the fundamental chemical structure of P2VN is largely unaffected by variations in sample preparation.

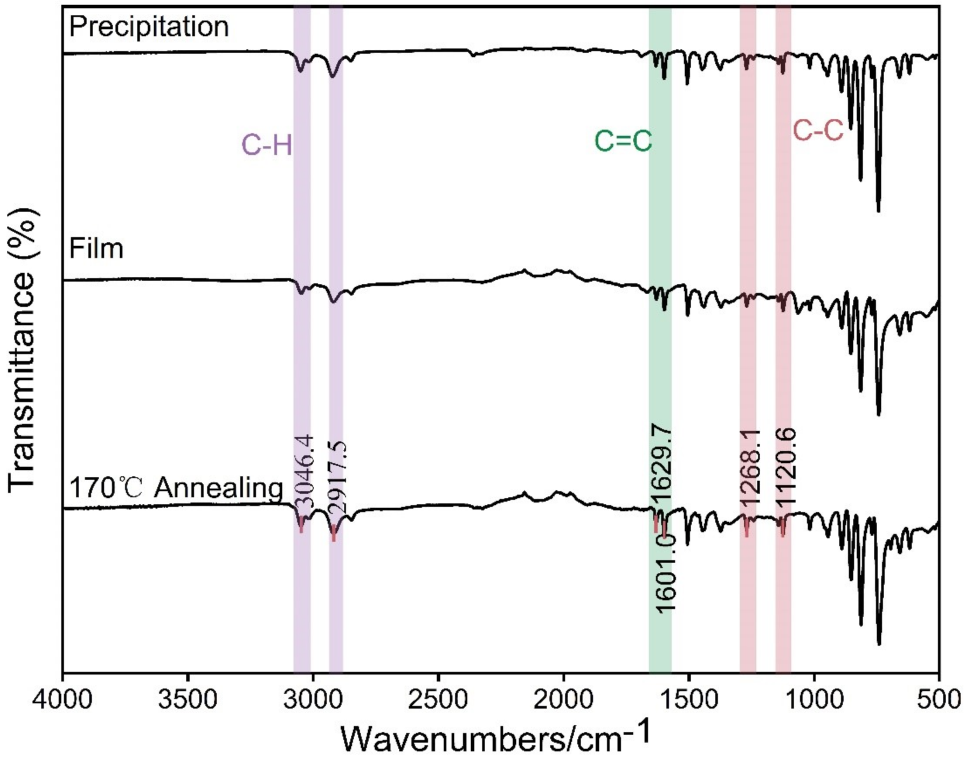

Fig S1. FT-IR spectra of P2VN samples snnealed at 170°C, film, and precipitated samples

2 ^{1}H-NMR spectra of P2VN in $CDCl_3$ and GPC traces of P2VN

As shown in Fig S2A, the strong and broad signals in this region correspond to the protons on the naphthalene rings of the P2VN repeating units. The peak at approximately 7.26 ppm corresponds to the residual solvent peak of $CDCl_3$, which is commonly observed in $CDCl_3$-based NMR spectra. The signals in the 7.0 – 8.5 ppm region are assigned to the aromatic protons of the naphthalene rings. The signals in the 1.5–3.0 ppm region are attributed to the protons of the polymer backbone (–CH_2–CH–). The sharp peak at 0 ppm is the internal standard TMS. As shown in Fig S2B, the number-average molecular weight (Mn) of the polymer was determined to be 103115 g/mol, and the polydispersity index was determined to be 1.09 by gel permeation chromatography (GPC) measurement.

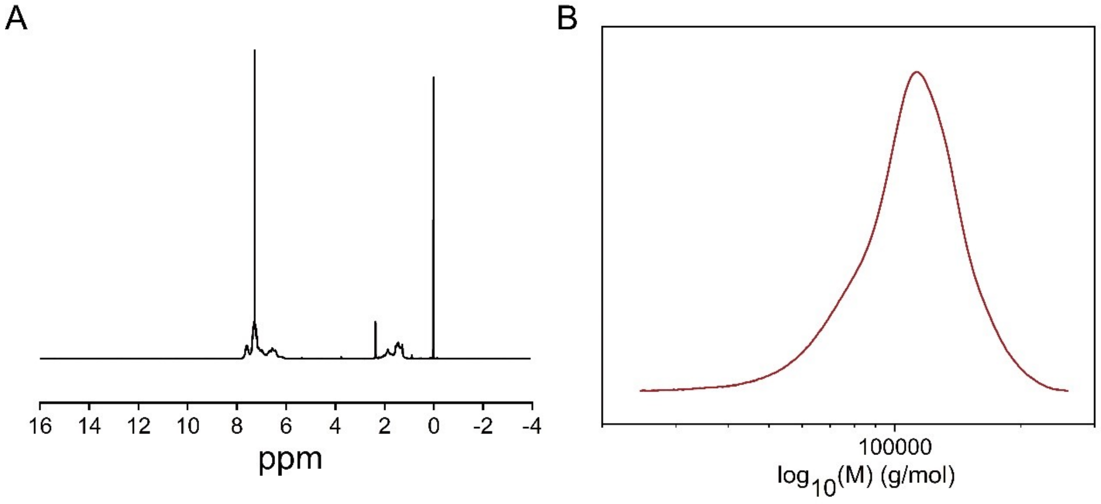

Fig S2. A. ^{1}H-NMR spectra of P2VN in $CDCl_3$; B. GPC traces of P2VN. Eluent: THF

3 Theoretical Calculations

3.1 The scanning and characterization of the potential energy surface in the conformational space of P2VN

All calculations were performed using Gaussian 09 and Multiwfn software packages.

Ground-state geometries were optimized at the B3LYP/6-31G(d) level with Grimme's DFT-D3 dispersion correction. Frequency analyses confirmed local minimum on potential energy surfaces. The potential energy surface (PES) scan was performed using the relaxed scanning approach. Excited states were calculated using time-dependent DFT (TD-DFT) at the same level of theory. Polymer systems were modeled using tetrameric oligomer approximations.

A potential energy surface (PES) scan was conducted to examine the changes in the C-C distance between naphthalene rings in the mesophase of C3-P2VN. As shown in Fig S3A, the total energy of the system decreases gradually with increasing interring C-C distance. A spatial interaction is observed between the naphthalene rings, with the system reaching its minimum energy at a distance of approximately 5.4 Å. Beyond this point, the energy increases steadily, and the spatial interaction between the naphthalene rings weakens, which adversely affects the luminescence properties of the system. Structural optimization was performed on configurations with C-C distances shorter than 5.4 Å. The optimized structures consistently exhibited a C-C distance of 4.7 Å. Thus, it can be inferred that when the C-C distance is less than 4.7 Å, spatial interactions between the naphthalene rings are enhanced, thereby promoting luminescence in the system.

PES scanning was also conducted to evaluate the dihedral angle changes of the branched naphthalene ring in C3-P2VN. The results, presented in Fig. S3B, reveal that the total energy of the system undergoes three consecutive "increase–decrease" cycles as the dihedral angle between the branched naphthalene ring and the main chain

enlarges. The global energy minimum corresponds to the most stable conformation, where spatial interactions between the naphthalene rings are optimized, thereby facilitating enhanced luminescence efficiency in the system.

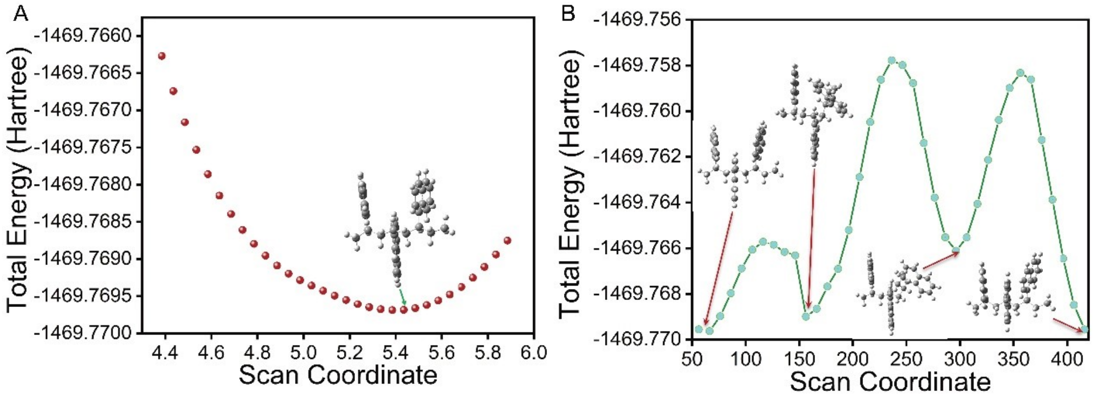

Fig S3 · (A) Potential energy surface scan: C-C bond length variation between naphthalene rings in the mesophase of C3-P2VN. (B) Potential energy surface scan: the dihedral angle variation in the branched-chain naphthalene ring of C3-P2VN.

3.2 Theoretical analysis of P2VN molecular properties and the photoluminescence spectra of P2VN in THF solutions at different concentrations

To establish the structure-property relationship between microstructural evolution and fluorescence intensity in P2VN, we performed:(i) DFT calculations for ground-state properties, (ii) FT-IR spectroscopy to probe excited-state characteristics, and(iii) UV-Vis spectroscopy for electronic transition analysis. All calculations were performed using Gaussian 09 and Multiwfn software packages. Simulation results demonstrate that the ground state and excited state FT-IR spectra exhibit nearly identical peak positions. Furthermore, comparative analysis with experimental infrared spectra reveals

superior accuracy in the characteristic peaks derived from theoretical simulations (Fig S4A and Fig S1).

This agreement not only validates the reliability of the experimental data but also corroborates the theoretical model's construction, particularly in the context of molecular conformational changes during fluorescence emission. As shown in Fig S4B, a prominent absorption peak emerges at approximately 310 nm, demonstrating poly(2-vinylnaphthalene)'s strong UV absorption at this wavelength. This observation is consistent with the experimental results presented in Fig 2A, B, and C. This optical behaviour stems from the material's molecular architecture, where the conjugated system in poly(2-vinylnaphthalene) provides abundant π-electrons, resulting in intense UV absorption typically associated with π-π* transitions.

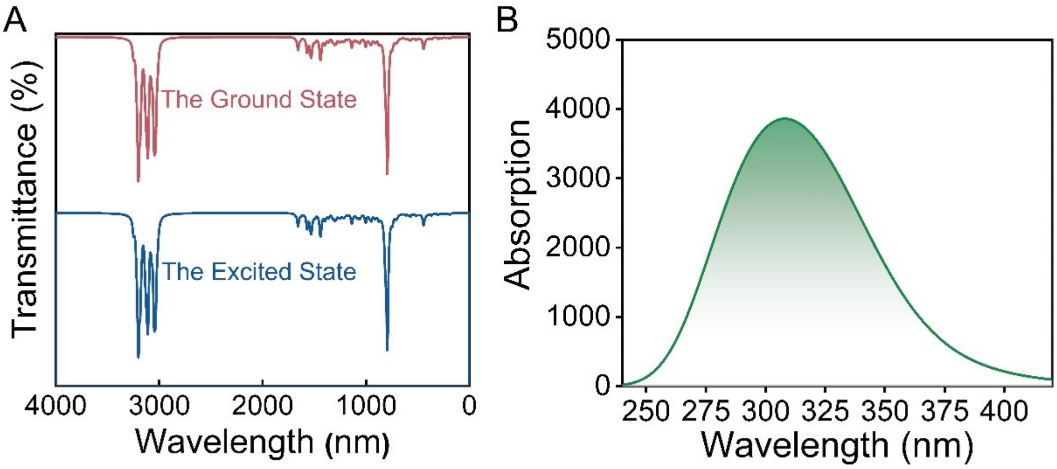

Fig S4. (A) Theoretical simulation of FT-IR spectra of P2VN ground state, excited state. (B). Theoretical simulation of UV-VIS spectra of P2VN.

As shown in Fig S5.A, the ordered stacking model exhibits a narrow characteristic peak, while the random stacking model yields a broadened peak,

consistent with our experimental solid-state absorption spectra (Fig 3A-C). As shown in Fig S5.B, the ordered structure of molecular chains is completely disrupted in solution, and the photoluminescence intensities of P2VN solutions at different concentrations are almost negligible.

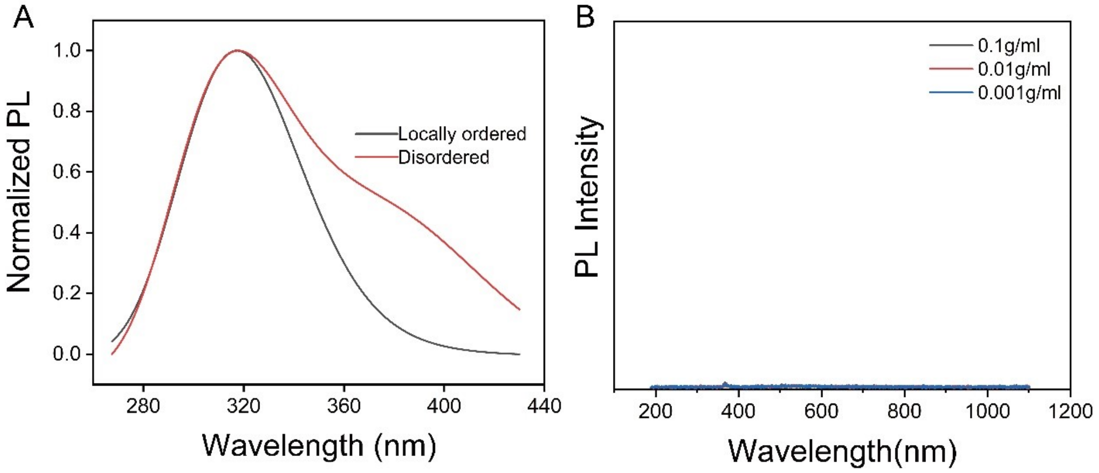

Fig S5. (A)Theoretical simulation of UV-VIS spectra of locally ordered and disordered P2VN; (B) Photoluminescence spectra of P2VN in THF solutions at different concentrations
